# Copper and Zinc Levels, Prevalence of Common Variants of Genes Involved in Their Metabolism and Psoriasis Disease

**DOI:** 10.3390/biomedicines13020529

**Published:** 2025-02-19

**Authors:** Tadeusz Dębniak, Piotr Baszuk, Ewa Duchnik, Karolina Rowińska, Magdalena Boer, Magdalena Kiedrowicz, Mariola Marchlewicz, Cezary Cybulski, Martyna Feherpataky, Róża Derkacz, Anna Dębniak, Emilia Rogoża-Janiszewska, Wojciech Marciniak, Marcin Lener, Jan Lubiński, Rodney J. Scott, Jacek Gronwald

**Affiliations:** 1 Department of Genetics and Pathology, International Hereditary Cancer Center, Pomeranian Medical University, 71-252 Szczecin, Poland; 2 Department of Clinical Genetics and Pathology, University of Zielona Góra, 65-046 Zielona Góra, Poland; 3 Department of Skin Diseases and Venerology, Pomorski Uniwersytet Medyczny, 72-010 Police, Poland; 4 Read-Gene, Grzepnica, 72-003 Dobra (Szczecińska), Poland; 5 Medical Genetics, School of Biomedical Sciences and Pharmacy, Callaghan, NSW 2308, Australia

**Keywords:** psoriasis, copper, zinc

## Abstract

**Background:** The pathogenesis of psoriasis is poorly understood. Increased reactive oxygen species (ROS) and lipid peroxidation are crucial in the inflammatory processes, including psoriasis. Thus, microelements, such as zinc and copper, may play a significant role in this disease’s development. **Methods:** Due to the paucity and inconsistency of literature data, we studied the levels of copper and zinc in blood and serum from 301 unselected psoriatic patients and 301 matched healthy controls and examined any associations among the microelements and clinical course or SOD2 (rs4880), CAT (rs1001179), GPX1 (rs1050450), and DMGDH (rs921943) DNA variants. **Results:** The mean blood copper levels were 864.94 µg/L and 907.24 µg/L, respectively, for controls and psoriasis patients (*p* < 0.001). The mean serum copper levels were 1,104.14 µg/L and 1191.72 µg/L, respectively, for controls and psoriasis patients (*p* < 0.001). The psoriasis risk was highest the among participants with the highest blood levels (>950.02 µg/L, OR: 2.36; 95% CI: 1.31–4.26; *p* = 0.004) and the highest serum concentrations (>1276.98 µg/L, OR: 3.08; 95% CI: 1.77–5.36; *p* < 0.001). The mean serum zinc levels were significantly lower (*p* < 0.001) among patients (910.87 µg/L) when compared to controls (979.68 µg/L). The mean blood zinc levels were not significantly different in cases and controls. Subjects with the lowest serum zinc levels (<843.68 µg/L) were affected more frequently (OR: 3.85; 95% CI: 2.24–6.60; *p* < 0.001). We found positive correlations between copper levels and PASI and inverse correlations of serum zinc levels with PASI and NAPSI scores. There were no associations between the levels of microelements and studied DNA variants. **Conclusions:** Our results support the thesis of an association between psoriasis onset and altered course of the disease with upset levels of copper and zinc. Future prospective studies might focus on optimization of the concentration of these trace elements for prophylaxis and to support the treatment of psoriasis.

## 1. Introduction

Psoriasis is one of the most frequent dermatological disorders, affecting 2% to 3% of the general Caucasian population [1]. It is a chronic skin disease characterized by hyperproliferation of keratinocytes, inflammation, and increased blood flow due to marked alteration of cytokine profiles and activation of skin immune cells. The pathogenesis of psoriasis is still not well understood. The interaction between molecular DNA alterations and different environmental factors, such as diet, stress, alcohol consumption, and smoking plays an important role. Increased reactive oxygen species (ROS) and lipid peroxidation are crucial in inflammatory processes. As such, they are seen in many dermatologic diseases, including psoriasis [2,3].

Microelements, such as zinc and copper, suppress free radicals through cascading enzyme systems [4]. Zinc and copper, being an integral part of around 1000 transcription factors and over 300 enzymes (such as copper/zinc superoxide dismutase) are involved in antioxidant and anti-inflammatory activity [5,6,7]. An imbalance between copper and zinc concentrations may result in altered antioxidant functions of many enzymes, and oxidative stress [8,9]. Recent studies of the role of microelements in the pathogenesis and treatment of psoriasis have shown controversial results and are still limited. While nonadherence is seen across all medical disciplines, the variety of treatment options available in dermatology warrants special attention [10]. There are only a few reports in the literature about copper and zinc levels in psoriasis. Most of them are based upon small numbers of patients and focus on serum concentrations only. Due to the paucity of literature data, it is justified to undertake research based upon examination of larger groups of cases and controls. Herein, in order to determine the impact of copper and zinc levels on psoriasis occurrence and its clinical course, we measured the concentration of both microelements in the serum and blood of 301 unselected patients with psoriasis and 301 healthy controls, and we evaluated any possible associations with clinical course such as length and severity of disease (measured by the PASI (Psoriasis Area and Severity Index), NAPSI (Nail Psoriasis Severity Index), and BSA (Body Surface Area) scales), treatment, family history, and the coincidence of cardiovascular, metabolic, or cancer diagnoses. This information can be a very important stepping stone for future studies focused on recurrent prophylaxis and support for the treatment of psoriasis by optimizing microelement concentrations. Finally, the simultaneous evaluation of the blood and serum levels of microelements in psoriatic patients can be important from a practical point of view—it can lead to optimization of routine examination of microelements in terms of selection of the material for testing (full blood or serum). Herein, in order to determine the impact of copper and zinc levels on psoriasis occurrence and its clinical course, we measured the concentration of both microelements in the serum and blood from 301 unselected patients with psoriasis plus 301 healthy controls, and we evaluated any possible associations with clinical course, such as length and severity of the disease (measured by the PASI, NAPSI, BSA scales), treatment, family history, and the coincidence of cardiovascular, metabolic or cancer diagnoses.

Among antioxidant enzymes suppressing oxidative stress, superoxide dismutases (SODs), catalase (CAT), and glutathione peroxidase 1 (GPx1) play primary roles in the detoxification of reactive oxygen species [11,12,13]. We have recently found that common variants of SOD2, CAT, and dimethylglycine dehydrogenase (DMGDH) genes may influence the levels of selenium, another microelement associated with psoriasis [14]. Herein, we additionally genotyped common variants of SOD2, CAT, GPX1, and DMGDH genes in order to evaluate their possible interactions with zinc and copper levels in psoriatic patients.

## 2. Materials and Methods

### 2.1. Patients

The study group consisted of 301 unselected psoriasis patients and 301 controls. The cases were recruited from the Clinic of Dermatology and Venerology at Pomeranian Medical University in Szczecin and other outpatient departments in Szczecin. In each case, the psoriasis diagnosis was confirmed by a dermatologist. The only exclusion criterium was age (<18). Among the psoriasis patients, there were 109 females (36%) and 192 males (64%). Clinical data were collected, including: psoriatic arthritis (yes/no), diabetes (yes/no), cardiovascular diseases (yes/no), liver diseases (yes/no), malignancies (yes/no), smoking (yes/no), alcohol consumption (yes/no), lactose intolerance (yes/no), gluten intolerance (yes/no), Hashimoto’s disease (yes/no), cholesterol (quantitative), HDL (quantitative), LDL (quantitative), glucose (quantitative), PASI (0–11/≥12), BSA (0–11/≥12), NAPSI (yes/no), BMI (quantitative), and PUVA/UVB treatment during last 6 months before study enrollment (yes/no) (Table 1).

A total of 301 sex- and age-matched subjects without psoriasis were enrolled into the control group for copper and zinc blood/serum level assessment. The control group was collected from the registry located at the Department of Genetics and Pathology of the Pomeranian Medical University in Szczecin.

During enrollment, the main goals of the study were explained, signed consent forms were obtained, genetic consultations were performed, and blood samples were collected for DNA, blood, and serum analyses.

### 2.2. Methods

Venous blood samples from all patients and controls were obtained in the morning (fasting) and frozen at −80 °C within 2 h following collection. Samples were sent within 2 days to the Department of Genetics and Pathology, where DNA was isolated from 5 mL of peripheral blood by standard methods. The rest of the material was used for evaluation of the concentration of microelements.

In each patient and healthy individual, DNA was genotyped for detection of the rs4880 variant in the SOD2 gene, rs1001179 in the CAT gene, rs1050450 in the GPX1 gene, and rs921943 in the DMGDH gene using a TaqMan assay (Applied Biosystems/Life Technologies) and the LightCycler Real-Time PCR 480 system (Roche Diagnostics GmbH). The primer and probe sequences are available upon request. Laboratory technicians were blinded to case–control status. The overall genotyping call rate was 99.3%.

Randomly selected samples were verified by Sanger sequencing.

Blood and serum concentrations of copper and zinc were analyzed in samples from 301 cases and 301 matched controls using the Inductively Coupled Plasma Mass Spectrometer (ICPMS, PerkinElmer, Waltham, MA, USA). Calibration standards were prepared by dilution of 10 mg/l Multi-Element Calibration Standard 3 (PerkinElmer Pure Plus, PerkinElmer Life and Analytical Sciences, Shelton, CT, USA) with reagent blanks consisting of a 0.65% solution of nitric acid (Merck, Darmstadt, Germany) and 0.002% Triton X-100 (PerkinElmer, Waltham, MA, USA). Calibration curves were created using four different concentrations: 0.1 µg/L, 0.5 µg/L, 1 µg/L, and 2 µg/L. Germanium (PerkinElmer Pure, PerkinElmer Life and Analytical Sciences, Shelton, CT, USA) was used as an internal standard and ClinChek^®^ Plasma Control Level I (Recipe, Darmstadt, Germany) was used as a reference material. Reference materials were measured after each of the six samples. If the difference between the reference material measurements was greater than 5%, the entire series was repeated. Each sample was measured in duplicate from different analytical runs. Prior to analysis, all samples were centrifuged (4000× *g*, 15 min) and the supernatant diluted 100 times with the reagent blank. Technical details, plasma operating settings, and mass-spectrometer acquisition parameters are available on request.

### 2.3. Statistical Analyses

Copper and zinc blood and serum levels were sorted in ascending order and assigned into one of four categories (Q1–Q4). Each category contains 25% of the observations from the entire study group. In each case, the reference category was the group with the lowest or the highest levels, depending on which contained the fewest disease cases.

To evaluate the potential relationship between blood/serum copper and zinc levels and psoriasis occurrence, univariable conditional logistic regression models were calculated based on the entire study group (n = 301) and on subgroups consisting of females (n = 109) and males (n = 192) separately.

To assess differences in copper and zinc blood levels among variants of individual genes, nonparametric Wilcoxon rank sum tests were conducted.

In order to estimate the presence of significant correlations between quantitative variables among psoriasis patients, Spearman’s rank correlation coefficients (ρ) were calculated. In all statistical analyses, results with test probability values of <0.05 were considered to be significant. All calculations and data management were performed in the R statistical environment [14].

## 3. Results

The mean blood copper levels were 864.94 µg/L and 907.24 µg/L for controls and psoriasis patients, respectively (*p* < 0.001). The mean serum copper levels were 1104.14 µg/L and 1191.72 µg/L for controls and psoriasis patients, respectively (*p* < 0.001).

Psoriasis was diagnosed over two times more frequently (OR: 2.36; 95% CI: 1.31–4.26; *p* = 0.004) among subjects with the highest blood copper levels (Q4: 950.02 µg/L–2039.40 µg/L) when compared to individuals with the lowest blood copper levels (Q1: 480.24 µg/L–787.94 µg/L). It was three times more frequently found (OR: 3.08; 95% CI: 1.77–5.36; *p* < 0.001) in participants with the highest serum copper levels (Q4: 1276.98 µg/L–2842.94 µg/L) compared to the baseline serum copper quartile (Q1: 493.84 µg/L–967.75 µg/L) (Table 2). Similar results to those obtained in the analysis of the entire study group were observed in subgroups of males and females—there was a significantly increased prevalence of disease among males with the highest copper blood concentrations (Q1 vs. Q4 OR = 3.63, 95% CI 1.50–8.81, *p* = 0.004) and copper serum levels (Q1 vs. Q4 OR = 2.65, 95% CI 1.38–5.10, *p* = 0.003), increased disease occurrence among females with high copper serum levels (Q1 vs. Q4 OR = 2.84, 95% CI 0.94–8.61, *p* = 0.065) and blood levels (Q1 vs. Q4 OR = 1.15, 95% CI 0.44–3.03, *p* = 0.8). The mean blood zinc levels were not significantly different (*p* = 0.4) in psoriasis patients (6277.87 µg/L) when compared to healthy individuals (6314.75 µg/L). The mean serum zinc levels were significantly lower (*p* < 0.001) among patients (910.87 µg/L) when compared to controls (979.68 µg/L).

We found no significant differences in psoriasis occurrence among participants with low and high blood zinc levels (Table 2). Similarly, we found no significant differences in disease occurrence among subgroups of males and females depending on their blood zinc concentrations (data available on request).

Subjects with the lowest serum zinc levels (Q1: 334.40 µg/L–843.68 µg/L) were affected over three times more frequently (OR: 3.85; 95% CI: 2.24–6.60; *p* < 0.001) when compared to participants with the highest serum zinc levels (Q4: 1037.52 µg/L–2036.51 µg/L) (Table 2). The disease occurred more frequently both among females with the lowest serum levels (Q1 vs. Q4 OR = 2.63, 95% CI 1.04–6.63, *p* = 0.041) and males with the lowest serum levels (Q1 vs. Q4 OR = 4.37, 95% CI 2.21–8.64, *p* <0.001).

**Figure 1:**
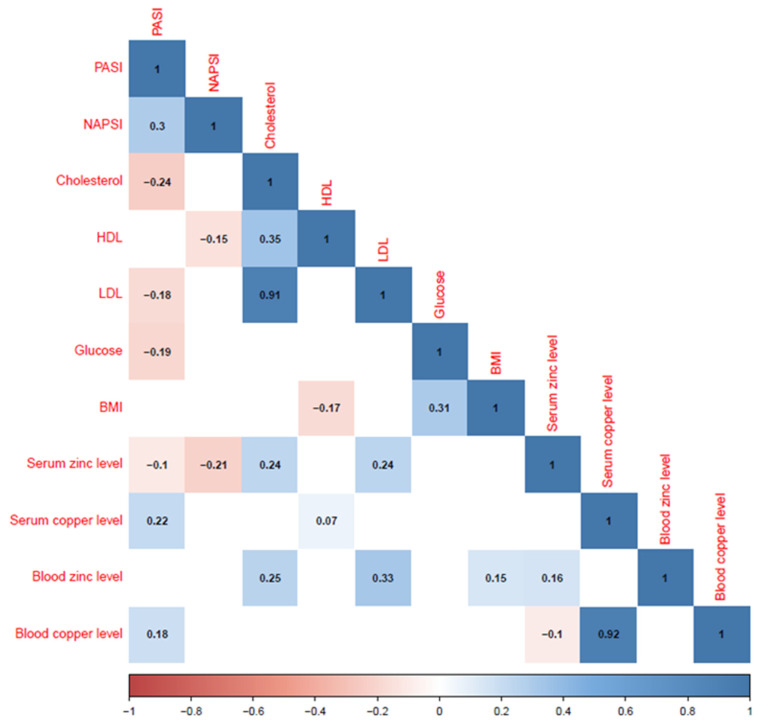
Spearman correlation coefficients for quantitative variables.

The evaluation of clinical data revealed that there was weak, positive collinearity between blood and serum copper levels and PASI (Spearmans ρ = 0.18 and ρ = 0.22, respectively) scores. In addition, serum zinc levels were negatively correlated with PASI (ρ = −0.1) and NAPSI (ρ = −0.21) scores. Serum zinc levels were also positively correlated with cholesterol (ρ = 0.24) and LDL (ρ = 0.24) levels (Figure 1).

There were no significant differences in the levels of blood/serum copper and zinc depending on the rs4880 variant in SOD2, rs1001179 in CAT, and rs921943 in DMGDH (Table 3).

## 4. Discussion

Literature data related to a possible association between copper, zinc and psoriasis are sparing and inconsistent.

Decreased levels of zinc among psoriatic patients was reported by Sheikh et al. (100 cases, 100 controls) [15]. No significant difference in the serum zinc concentration was found by Wacewicz et al. (60 cases, 58 controls) [7], Ala et al. (25 patients, 25 healthy individuals) [16], and Kreft et al. (88 cases, 22 controls) [17]. Increased zinc concentrations were observed by Butnaru et al. (25 cases, 50 controls) [18] and Elhaddad et al. (60 patients, 21 healthy adults) [19]. In majority of studies, elevated copper concentrations were reported among patients with psoriasis [7,15,16,18,19,20]. However, according to Bhatnagar et al., the level of this trace element is decreased in patients with psoriasis [21].

Herein, we found that both blood and serum mean copper levels were higher among patients when compared to healthy controls. The psoriasis occurrence among study participants was approximately two to three times higher in the subgroups with the highest blood and serum copper levels. Similar results to those obtained in analyzing the entire study group were observed in the male and female subgroups. Interestingly, our results also suggest that higher levels of copper are also associated with more severe course of psoriasis as measured by PASI scores.

It seems that we can confirm a link between copper and psoriasis regardless of the biological material we used. Due to fact that blood measurements are easier to perform in terms of logistics, this indicates that blood can be used in order to create an optimal algorithm for evaluation of copper levels in patients with this disease. We found decreased serum zinc levels among psoriatic patients and a significant association between low serum zinc levels and disease onset as well as disease worsening as measured by PASI and NAPSI scores. We were unable to demonstrate any association between blood zinc concentrations and disease occurrence or psoriasis course.

It appears that copper and zinc associations with psoriasis are moderate and weaker than that of another microelement—selenium. Psoriasis risk never reached the values seen during the selenium evaluation [22], and, in case of zinc, the apparent relationship additionally depended on the biological material used. We were unable to demonstrate any link between copper and zinc levels and occurrence of other abnormalities in our cohort of cases, such as cardiovascular disorders, metabolic diseases, Hashimoto’s disease, lactose/gluten intolerance, or malignancies. However, it cannot be ruled out that this finding was due to the small sizes of the subgroups when stratified according to clinical parameters, thus leading to a lower statistical power of the study. We were only able to demonstrate a positive correlation between serum zinc levels and cholesterol and LDL concentrations, which needs to be evaluated in additional studies.

Herein, we found no correlations between the common variants of SOD2, CAT, GPX1, and DMGDH and concentrations of the studied microelements, which strongly suggests that these DNA variants have no impact on copper and zinc metabolism. A major limitation of this study was the small size of the study groups, which reduces the statistical power of the study.

## 5. Conclusions

Our results support the thesis of an association between psoriasis onset and an altered course of disease in the presence of upset levels of copper and zinc. Future prospective studies might focus on optimization of the concentration of these trace elements for prophylaxis and to support the treatment of psoriasis.

## Figures and Tables

**Table 1:** Clinical characteristics of psoriasis patients.

Variables	Psoriasis Patients, n = 301	Unknown
**Sex**		
Females	109 (36%)	
Males	192 (64%)	
**Psoriatic Arthritis**		13
No	204 (71%)	
Yes	84 (29%)	
**Diabetes**		15
No	262 (92%)	
Yes	24 (8.4%)	
**Cardiovascular Diseases**		17
No	224 (79%)	
Yes	60 (21%)	
**Liver Diseases**		18
No	270 (95%)	
Yes	13 (4.6%)	
**Malignancies**		18
No	270 (95%)	
Yes	13 (4.6%)	
**Smoking**		14
No	184 (64%)	
Yes	103 (36%)	
**Alcohol Consumption**		20
No	153 (54%)	
Yes	128 (46%)	
**Lactose Intolerance**		58
No	229 (94%)	
Yes	14 (5.8%)	
**Gluten Intolerance**		58
No	232 (95%)	
Yes	11 (4.5%)	
**Hashimoto’s Disease**		59
No	228 (94%)	
Yes	14 (5.8%)	
**Cholesterol**	86.45–747.20 (191.76)	42
**HDL**	23.60–103.99 (54.23)	48
**LDL**	26.09–230.53 (123.67)	47
**Glucose**	55.60–210.60 (99.17)	38
**PASI**		19
0–11	143 (51%)	
≥12	139 (49%)	
**BSA**		20
0–9	97 (35%)	
≥10	184 (65%)	
**BMI**	17.71–45.58 (28.07)	53
**PUVA/UVB during the last 6 months**		84
No	213 (98%)	
Yes	4 (1.8%)	
**NAPSI**	0.00–160.00 (11.78)	26

n (%); Range (Mean).

**Table 2:** Psoriasis occurrence depending on copper and zinc blood/serum levels.

Variables	Controls, n = 301	Cases, n = 301	OR	95% CI	*p*
**Blood copper level**					
Q1 (reference): 480.24–787.94 (733.13)	69 (27%)	68 (23%)	-	-	
Q2: 788.28–860.16 (825.70)	75 (29%)	62 (21%)	0.78	0.48–1.29	0.3
Q3: 861.28–949.83 (906.67)	68 (26%)	69 (24%)	1.14	0.67–1.94	0.6
Q4: 950.02–2039.40 (1084.12)	45 (18%)	92 (32%)	2.36	1.31–4.26	0.004
Misisng	44	10			
**Serum copper level**					
Q1 (reference): 493.84–967.7(871.67)	80 (27%)	70 (24%)	-	-	
Q2: 970.97–1101.08 (1038.18)	95 (32%)	54 (18%)	0.59	0.36–0.97	0.038
Q3: 1101.28–1275.26 (1188.01)	75 (25%)	74 (25%)	1.34	0.82–2.19	0.2
Q4: 1276.98–2842.94 (1491.04)	51 (17%)	99 (33%)	3.08	1.77–5.36	<0.001
Missing	0	4			
**Blood zinc level**					
Q4 (reference): 6748.81–8528.76 (7238.44)	56 (24%)	77 (26%)	-	-	
Q1: 3798.91–5811.49 (5371.72)	68 (29%)	64 (22%)	0.57	0.32–1.00	0.05
Q2: 5812.54–6285.21 (6084.54)	58 (24%)	74 (25%)	0.79	0.47–1.31	0.4
Q3: 6285.50–6740.52 (6490.81)	56 (24%)	76 (26%)	1.02	0.61–1.72	>0.9
Missing	63	10			
**Serum zinc level**					
Q4 (reference): 1037.52–2036.51 (1148.09)	88 (29%)	62 (21%)	-	-	
Q1: 334.40–843.68 (746.25)	43 (14%)	107 (36%)	3.85	2.24–6.60	<0.001
Q2: 843.77–952.02 (895.37)	76 (25%)	73 (25%)	1.53	0.93–2.52	0.091
Q3: 952.08–1037.26 (992.30)	94 (31%)	55 (19%)	0.79	0.47–1.32	0.4
Missing	0	4			

n (%); min-max (mean).

**Table 3:** Selenium blood levels and *SOD2*, *CAT*, *GPX1*, and *DMGDH* variants.

	*SOD2*			*CAT*			*GPX1*			*DMGDH*		
Variable	CC	nonCC	*p*	CC	nonCC	*p*	CC	nonCC	*p*	CC	nonCC	*p*
**Blood zinc level**	3159.15–8780.43 (6266.31)	3798.91–8528.76 (6273.34)	0.9	3159.15–8675.61 (6264.50)	3986.47–8780.43 (6283.12)	>0.9	3798.91–8514.34 (6253.71)	3159.15–8780.43 (6287.74)	0.8	3798.91–8264.01 (6260.13)	3159.15–8780.43 (6281.28)	0.7
**Blood copper level**	649.30–2039.40 (878.50)	13.45–1739.28 (872.09)	0.6	13.45–1739.28 (870.38)	480.24–2039.40 (879.15)	0.8	516.80–1598.20 (873.08)	13.45–2039.40 (874.38)	0.6	480.24–1739.28 (873.63)	13.45–2039.40 (873.88)	0.6
**Serum zinc level**	334.40–1794.44 (965.37)	508.63–2036.51 (944.50)	0.3	508.63–2036.51 (949.74)	334.40–1807.81 (950.74)	0.9	508.63–2036.51 (954.99)	334.40–1807.81 (945.57)	0.5	509.61–1471.26 (938.09)	334.40–2036.51 (960.73)	0.12
**Serum copper level**	639.58–2704.35 (1149.35)	493.84–2842.94 (1144.86)	0.9	526.04–2520.52 (1140.22)	493.84–2842.94 (1155.81)	>0.9	526.04–2117.01 (1123.94)	493.84–2842.94 (1166.71)	0.14	493.84–2842.94 (1146.08)	526.04–2704.35 (1146.06)	0.7

min-max (mean).

## Data Availability

The datasets used and/or analyzed during the current study are available from the corresponding author on reasonable request.
